# c‐Myc promotes lymphatic metastasis of pancreatic neuroendocrine tumor through VEGFC upregulation

**DOI:** 10.1111/cas.14717

**Published:** 2020-11-24

**Authors:** Tsung‐Ming Chang, Pei‐Yi Chu, Wen‐Chun Hung, Yan‐Shen Shan, Hui‐You Lin, Kuo‐Wei Huang, Jeffrey S. Chang, Li‐Tzong Chen, Hui‐Jen Tsai

**Affiliations:** ^1^ National Institute of Cancer Research National Health Research Institutes Tainan Taiwan; ^2^ Department of Pathology Show Chwan Memorial Hospital Changhua Taiwan; ^3^ School of Medicine College of Medicine Fu Jen Catholic University New Taipei City Taiwan; ^4^ School of Pharmacy College of Pharmacy Kaohsiung Medical University Kaohsiung Taiwan; ^5^ Department of Surgery National Cheng Kung University Hospital Tainan Taiwan; ^6^ Institute of Clinical Medicine National Cheng Kung University Tainan Taiwan; ^7^ Department of Oncology College of Medicine National Cheng Kung University Hospital National Cheng Kung University Tainan Taiwan; ^8^ Department of Internal Medicine Kaohsiung Medical University Hospital Kaohsiung Medical University Kaohsiung Taiwan; ^9^ Institute of Molecular Medicine National Cheng Kung University Tainan Taiwan

**Keywords:** c‐Myc, lymphangiogenesis, mTOR, pancreatic neuroendocrine tumor, vascular endothelial growth factor C

## Abstract

Pancreatic neuroendocrine tumor (pNET) is a pancreatic neoplasm with neuroendocrine differentiation. pNET in early stage can be treated with surgical resection with long‐term survival, whereas the prognosis of pNET with locoregional or distant metastasis is relatively poor. Lymphangiogenesis is essential for tumor metastasis via the lymphatic system and may overhead distant metastasis. c‐Myc overexpression is involved in tumorigenesis. The role of c‐Myc in lymphangiogenesis is unclear. In this study, we evaluated the mechanism and effect of c‐Myc on lymphangiogenesis of pNET via interaction of lymphatic endothelial cells (LECs) and pNET cells. Lymph node metastasis was evaluated in pNET xenograft mice. Potential target agents to inhibit lymph node metastasis were evaluated in an animal model. We found that vascular endothelial growth factor C (VEGFC) expression and secretion was increased in pNET cell lines with c‐Myc overexpression. c‐Myc transcriptionally upregulates VEGFC expression and the secretion of pNET cells by directly binding to the E‐box of the VEGFC promoter and enhances VEGF receptor 3 phosphorylation and the tube formation of LECs. c‐Myc overexpression is associated with lymph node metastasis in pNET xenograft mice. Combinational treatment with an mTOR inhibitor and c‐Myc inhibitor or VEGFC‐neutralizing chimera protein reduced lymph node metastasis in the mice with c‐Myc overexpression. The mTOR inhibitor acts on lymphangiogenesis by reducing VEGFC expression in pNET cells and inhibiting the tube formation of LECs. In conclusion, mTOR and c‐Myc are important for lymphangiogenesis of pNET and are potential therapeutic targets for prevention and treatment of lymph node metastasis in pNET.

## Graphical abstract

c‐Myc promotes lymph node metastases of pancreatic neuroendocrine tumors via upregulation of vascular endothelial growth factor C (VEGFC). Combined targeting of mTOR with c‐Myc or VEGFC is a potential therapy for the treatment of pNET.

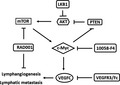

## 1 INTRODUCTION

Pancreatic neuroendocrine tumor (pNET) is a rare histologic subtype of pancreatic cancer with the expression of neuroendocrine markers. It accounts for approximately 3% of all pancreatic cancers in Taiwan.^1^ The incidence of pNET has significantly increased in the USA and Taiwan in the recent decade.^1^, ^2^ Although the localized pNET has a long‐term survival (10‐year survival rate of 62%‐79%), pNET with distant metastasis has a 10‐year survival rate approximating 20% according to the Surveillance, Epidemiology, and End Results (SEER) in the USA.^3^ There are many factors reported to be associated with the overall survival of pNET, such as tumor grade, Ki‐67 index, mitotic rate, age, sex, lymph node status, liver metastasis, and bone metastasis.^4^, ^5^, ^6^, ^7^ Lymph node metastasis is an important prognostic factor for tumor recurrence and outcome of cancers.^8^ The association of lymph node status with postoperative recurrence or survival in pNET is controversial.^9^, ^10^, ^11^, ^12^, ^13^ However, Jiang et al^14^ have shown that the presence of lymph node metastases was significantly associated with decreased disease‐free survival (hazard ratio = 3.995, 95% confidence interval: 1.585‐10.06, *P* = 0.003) for 100 pNET patients who underwent surgical resection. Krampitz et al^15^ have shown that pNET patients with lymph node metastases alone have a short time to develop liver metastases and have decreased disease‐related survival according to a prospective database analysis from the National Institute of Health and Stanford University Hospital.

The metastasis of tumor cells occurs through lymphatic and blood vessels. Lymphatic vessels undergoing dynamic changes, such as lymphangiogenesis and lymphatic vessel remodeling, may facilitate metastasis.^8^ There are many molecules associated with lymphangiogenesis, including vascular endothelial growth factor C (VEGFC)/D, VEGFA, angiopoietins, growth factors (hepatocyte growth factor (HGF), fibroblast growth factor, epidermal growth factor (EGF), platelet‐derived growth factor (PDGF), and insulin‐like growth factor), inflammatory cytokines, etc.^16^ Among them, the VEGFC/VEGF receptor 3 (VEGFR3) and VEGFD/VEGFR3 axis is known as a major driver of lymphangiogenesis.^8^, ^16^ Lymph node metastasis was shown to be associated with the recurrence and disease‐related survival of pNET.^14^, ^15^ However, the mechanism of lymph node metastasis in pNET was not well understood. We have identified a high percentage of c‐Myc overexpression in pNET via Phosphatase and tensin homolog (PTEN)/liver kinase B1 (LKB1)‐dependent and PTEN/LKB1‐independent mechanisms previously.^17^ In this study, we investigated the association of c‐Myc and lymph node metastasis in pNET, delineated the regulatory mechanism, and identified the potential targeted agents against lymph node metastasis in pNET.

## 2 MATERIALS AND METHODS

This study was approved by the Institutional Review Board of the National Health Research Institutes.

### 2.1 Cell lines, plasmids, and reagents

QGP‐1, a human pNET cell line, was purchased from the Japanese Collection of Research Bioresources (JCRB, Tokyo, Japan). NIT‐1, a mouse pNET cell line, was purchased from the Bioresource Collection and Research Center (BCRC, Hsinchu, Taiwan). QGP‐1 cells were cultured in RPMI‐1640 (Hyclone) medium containing 10% fetal calf serum and antibiotics. NIT‐1 cells were cultured in F12‐Kaighn's (Gibco) medium containing 10% fetal calf serum and antibiotics. The QGP‐1 cell line was sent to the Center for Genomic Medicine of the National Cheng Kung University for genotyping in June 2016, and the result showed the same short tandom repeat PCR DNA profiles as those in the JCRB database. Human dermal lymphoepithelial cells (HDLECs), which was kindly provided by Dr Wen‐Chun Hung (National Health Research Institutes, Tainan, Taiwan), were obtained from Promo Cell and cultured in endothelial cell growth medium MV2 (EGM‐MV2) according to manufacturer's instruction. The passage of these cell lines used in this study was <15. All cell lines were mycoplasma‐free. Human and mouse c‐Myc expression plasmids were purchased from Addgene. ShRNA‐targeting c‐Myc was obtained from the National RNAi Core Facility of Academia Sinica (Taipei, Taiwan). Recombinant human VEGFR3‐Fc chimera protein was purchased from R&D Systems and Sino Biological and reconstituted in sterile PBS. RAD001 was purchased from Calbiochem and reconstituted in DMSO. The c‐Myc inhibitor, 10058‐F4, was purchased from Selleckchem and reconstituted in 2% DMSO and corn oil.

### 2.2 Plasmid transfection and lentiviral infection

Human c‐Myc overexpression and c‐Myc knockdown by shRNA were conducted by a lentiviral infection system according to Addgene’s instruction. Briefly, lentiviral suspension was produced from 293T cells by transfecting expressing vector, packaging plasmid, and enveloping plasmid. We added lentiviral suspension to infect pNET cells in growth medium containing polybrene for 24 hours. Then the growth medium of the pNET cells was removed, and fresh growth medium was added to the lentiviral vector–infected pNET cells for another 24 hours. Then, puromycin was added to the growth medium of pNET cells for selecting lentiviral vector–infected cells to obtain stable expression clones. Mouse c‐Myc overexpression and VEGFC promoter‐luciferase plasmids transfection were performed by using Turbofect transfection reagent (Thermo) according to the manufacturer's instruction. Briefly, 1 µg of DNA and 2 µL of transfection reagent were mixed in serum‐free growth medium and added into a 70%‐90% confluent cell layer. Transgene expression of the cells was measured after incubation for 24‐48 hours.

### 2.3 RNA extraction and RT‐PCR

Total RNA was isolated from control or c‐Myc–overexpressed QGP‐1 and NIT‐1 cells by using a total RNA mini kit (Geneaid), and a high‐capacity cDNA reverse transcription kit (Applied Biosystems Inc) was used to perform reverse transcription according to the manufacturer's protocol. VEGFC expression was examined by using SYBR green PCR master mix, and β‐actin was used as an internal control to check the efficiency of cDNA synthesis and PCR amplification. The primers used were: VEGFC‐forward, 5′‐CAGTTACGGTCTGTGTCCAGTGTAG‐3′; VEGFC‐reverse, 5′‐GGACACACATGGAGGTTTAAAGAAG‐3′; β‐actin‐forward, 5′‐GCTGTGCTACGTCGCCCT‐3′; and β‐actin‐reverse, 5′‐ AAGGTAGTTTCGTGGATGCC‐3′. Mouse VEGFC‐forward, 5′‐CCAGCACAGGTTACCTCAGCAA‐3′; Mouse VEGFC‐reverse, 5′‐TAGACATGCACCGGCAGGAA‐3′; Mouse actin‐forward, 5′‐CACTGTCGAGTCGCGTCC‐3′; and Mouse actin‐reverse, 5′‐TCATCCATGGCGAACTGGTG‐3′. After reaction, the PCR products were separated on a 3% 0.5 × Tris‐acetate‐EDTA agarose gel and visualized under a UVP biospectrum image system.

### 2.4 Western blot

Whole cell lysates were harvested in lysis buffer. Equal amount of proteins was subjected to SDS‐PAGE. Proteins were transferred onto a PVDF membrane, and the blots were incubated with different primary antibodies, including VEGFC (1:1000), VEGFR3 (1:1000), TERT (1:1000), and GAPDH (1:5000) from Santa Cruz; phosphor‐VEGFR3 (1:1000) from Cell Applications; c‐Myc (1:1000) and E2F1 (1:1000) from Abcam; and acetyl Histone 3 (K9/K14) (1:1000), Histone 3 (1:1000), and MAX (1:1000) from GeneTex. Enhanced chemiluminescence reagents were used to depict the protein bands on the membrane and visualized by a UVP biospectrum image system.

### 2.5 VEGFC promoter activity assay

VEGFC promoter‐luciferase plasmids were kindly provided by Dr Wen‐Chun Hung. Briefly, a serial of plasmids with deletion on the VEGFC promoter region (−1046/+38, −439/+38 and −185/+38) from the translational start site of the VEGFC gene were amplified by PCR from the genomic DNA. Three DNA fragments were subcloned into the luciferase reporter gene vector pGL3 to yield the luciferase reporter construct. In addition, an E‐box mutant (TACGTG instead of CACGTG) of the VEGFC promoter (−1046/+38) was created by site‐directed mutagenesis (Promega) using a pair of primer (forward: 5′‐CCCTGGACCACGTACAGCGGGGAGAAA‐3′, reverse: 5′‐TTTCTCCCCGCTGTACGTGGTCCAGGG‐3′; Figure S1). Control or c‐Myc–overexpressed 293T cells were seeded into 12‐well plates and transfected with serial VEGFC promoter‐luciferase plasmids (VEGFC‐1046/+38, VEGFC‐439/+38, and VEGFC‐185/+38). The seeded cells were harvested 24 hours later and the luciferase activities of the cells in each condition were measured by luciferase assay system (Promega Corporation) and detected by CentroLIApc LB 962. Relative luciferase unit (RLU) was normalized by protein concentration in cell lysates.

### 2.6 Chromatin immunoprecipitation assay

Chromatin immunoprecipitation (ChIP) assay was performed as described previously.^18^ Briefly, control, c‐Myc–overexpressed, and c‐Myc–knocked‐down QGP‐1 cells were fixed with 1% formaldehyde at 37°C for 10 minutes and washed by cold PBS. The cells were harvested and lysed with RIPA buffer, and the collected cell lysate was sonicated to shear DNA to an average fragment size of 500*‐*1000 bp. After sonication, the cell lysate was treated with RNase A to remove RNA and with protease K to cleave peptide bonds. Anti‐c‐Myc (2 μg/mL) and anti‐Rabbit IgG (negative control) (2 μg/mL) antibodies were used for precipitating the protein/DNA complex. DNA fragments were collected and subjected to PCR amplification by using the primers specific for the detection of the −987 to −865 VEGFC promoter region which contained E‐box sequence. The sequences for the primers are forward: 5′‐GGGAGGGAGGACAAGAACTC‐3′ and reverse: 5′‐GACCGGCTTTAGAGGTGATG‐3′.

### 2.7 Immunoprecipitation assay

Control, c‐Myc–overexpressed, and c‐Myc–knocked‐down QGP‐1 cells were harvested and lysed by RIPA buffer. The cell lysate was incubated with anti‐c‐Myc (1 μg/mL) and anti‐rabbit IgG (negative control; 1 μg/mL) antibodies at 4°C overnight. c‐Myc–associated protein complex was pulled down by protein A/G and subjected to Western blot.

### 2.8 Measurement of VEGFC secretion

QGP‐1 and NIT‐1 cells transfected with vector control or c‐Myc overexpression plasmids were cultured in serum‐free growth medium for 24 hours. Then, the growth medium was collected and concentrated 30‐fold by an *amicon ultra* centrifugal filter device (Merck). The concentrated secretion from the cells was used to evaluate the amount of VEGFC secretion from the cells by Western blot.

### 2.9 Tube formation assay of lymphoepithelial cells (LECs)

Growth factor–reduced Matrigel (BD Biosciences) was coated to each well of prechilled channel slide and incubated at 37°C for 30 minutes for gelation. A total of 6000 LECs were seeded into each well and stimulated by conditional medium from control or c‐Myc–overexpressed QGP‐1 cells with or without VEGFR3‐Fc chimera protein. Tube formation was observed 4 hours later, and the images were captured by using a Leica DMI 4000 phase‐contrast microscope (Leica Microsystems) with contrast objective. Tube formation ability was analyzed by using the NIH Image J software.

### 2.10 Animal study

NOD‐SCID (6‐ to 8‐week‐old) male mice were obtained from LASCO and housed under specific pathogen–free conditions according to the guidelines of the Animal Care Committee at the National Health Research Institutes, Taiwan. 1 × 10^7^ QGP‐1 (control or c‐Myc–overexpressed) cells mixed with Matrigel (BD Biosciences) in 0.1 mL were injected subcutaneously into each mouse. The tumor volume was measured by caliper measurements and calculated as length (mm) × width^2^ (mm) × (π/6).^19^ The mice harboring QGP‐1/c‐Myc tumor were orally administered RAD001 (5 mg/kg) or intratumorally injected with 10058‐F4 (30 mg/kg) or VEGFR3/Fc chimera protein (0.5 mg/kg), either alone or in combination for 2 weeks (5 days treatment and 2 days rest per week) after the tumors had developed to approximately 40‐50 mm^3^. Tumor volumes were measured twice a week from the initiation of treatment for 2 weeks, and then the mice were sacrificed by CO_2_ exposure in home cage. All procedures of animal experiments were carried out in the laboratory of an animal center.

### 2.11 Pathological examination

Tumor and proximal (inguinal) lymph node from each mouse were harvested and fixed totally in 10% formalin for 24 hours at room temperature, washed in PBS, and embedded in paraffin. Four‐micrometer sections of paraffin‐embedded tissue were stained with hematoxylin and eosin (H&E stain). The anti‐LYVE1 antibody (ab 14917) was purchased from Abcam. The anti‐VEGFC (GTX 113574) was obtained from GeneTex. The anti‐Ki‐67 antibody (GM 010) was purchased from Genemed. The specimens were embedded in paraffin, cut into 4‐µm‐thick sections, attached to slides, and coated with poly‐l‐lysine. After deparaffinizing and rinsing with 10 mmol/L Tris‐HCl (pH 7.4) and 150 mmol/L sodium chloride, the slides were treated with methanol and 3% hydrogen peroxide, and then placed in a 100°C heating chamber for 20 minutes in 10 mmol/L citrate buffer. After that, they were incubated with LYVE1 (1:200), VEGFC (1:400), and Ki‐67 (1:500) antibody solutions for 1 hour. The slides were then washed with phosphate‐buffered saline, and LYVE1, VEGFC, and Ki‐67 antibodies were detected using the EnVision Detection Systems, Peroxidase/DAB, Rabbit/Mouse kit (Dako). Finally, the slides were analyzed under a microscope (BX50; Olympus). The negative samples and the control group were processed without the primary antibody.

The H&E sections, LYVE1, VEGFC, and Ki‐67 expression results were evaluated by a board‐certified pathologist (Dr Pei‐Yi Chu). LYVE1 expression was used to detect the intratumor lymphatic vessel density (LVD). The whole‐slide intratumor LYVE1 immunostain was evaluated to search for the hot‐spot areas of higher LYVE1 expression. Ten high‐power field areas (with 200× magnification under microscope) in the adjacent areas of hot spot with higher LYVE1 expression were evaluated to count the total number of lymphatic vessels. The number of intratumor LVD was defined as “the number of lymphatic vessels/10 high‐power field (200×) in the tumor areas.” The VEGFC expression results were evaluated and the scoring system was defined by two aspects: staining intensity (0, 1+, 2+, and 3+) and percentage of positive cells (0%‐100%). The expression of VEGFC was classified as high expression when the staining intensity was 2+ and/or 3+, whereas the expression of VEGFC was classified as low when the staining intensity was 0+ and/or 1+ without 2+ or 3+.

Ki‐67 expression results were carried out by scanning the whole slide without counting individual nuclei cells under a microscope with a 10× ocular and a 10× objective (100× total magnification). The percentage of tumor Ki‐67 expression (%) was estimated as the percentage of the tumor cells with Ki‐67 expression among the viable tumor cells.

### 2.12 Statistical analysis

The difference of relative luciferase activity and relative tube formation was analyzed by *t*‐test using EXCEL (Microsoft). The comparison for the difference of tumor growth and lymph node metastasis in the animal study was analyzed by Wilcoxon’s rank‐sum test and Fisher's exact test, respectively, using SAS (SAS Institute Inc). The correlation between VEGFC expression or LVD and c‐Myc overexpression in xenograft mice was analyzed by Fisher's exact test and Wilcoxon’s rank‐sum test, respectively.

## 3 RESULTS

### 3.1 c‐Myc positively correlates with VEGFC expression in pNET cells

**FIGURE 1:**
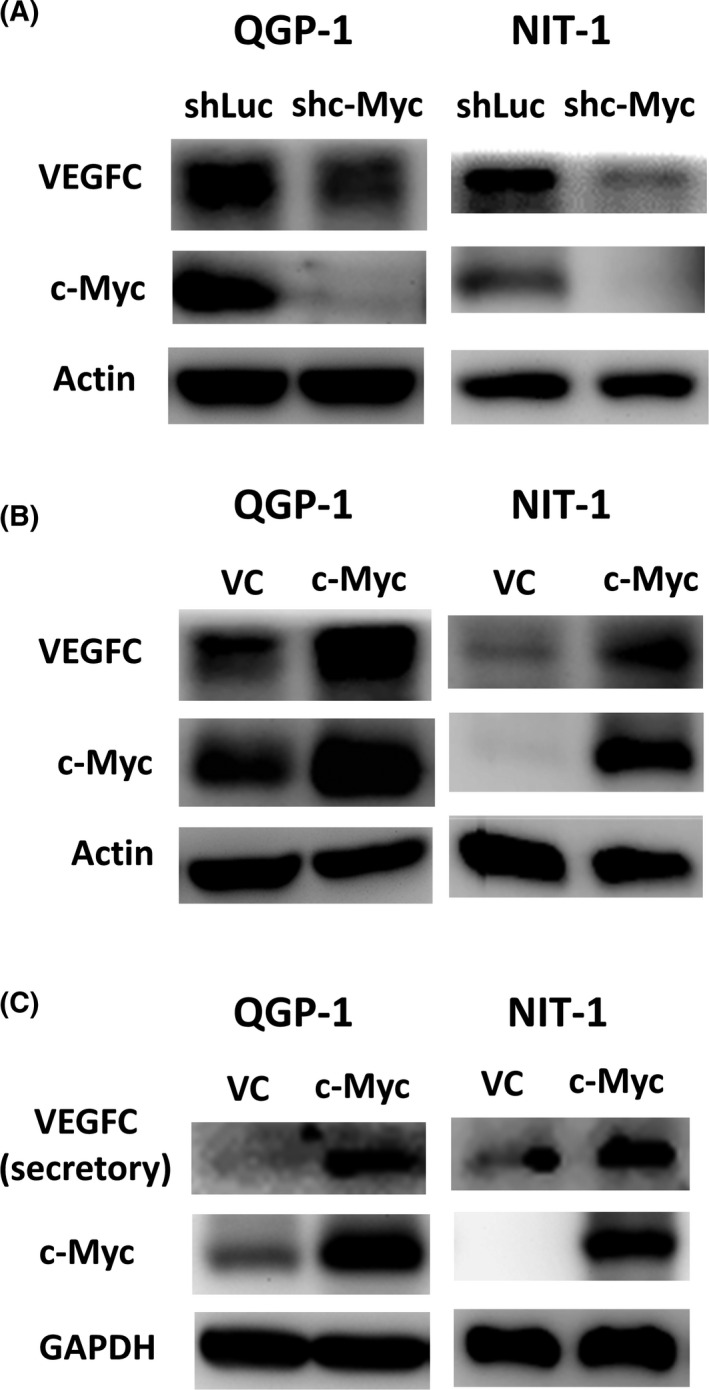
c‐Myc positively correlates with vascular endothelial growth factor C (VEGFC) expression in pancreatic neuroendocrine tumor (pNET) cells. A, VEGFC expression in QGP‐1 and NIT‐1 cells with and without knockdown of c‐Myc. B, VEGFC expression in QGP‐1 and NIT‐1 cells with and without overexpression of c‐Myc. C, Secretory amount of VEGFC in QGP‐1 and NIT‐1 cells with and without overexpression of c‐Myc. VC, vector control

We have found that enlarged lymph node was noted in the xenograft mouse model of pNET cells (QGP‐1) with PTEN and/or LKB1 loss (data not shown), which activate c‐Myc.^17^ In order to know whether c‐Myc activation is associated with lymph node metastasis in pNET, we evaluated the effect of c‐Myc on VEGFC expression in pNET cells (QGP‐1 and NIT‐1). Figure 1A shows that the expression of VEGFC was decreased in both cell lines with c‐Myc knockdown. By contrast, Figure 1B shows that VEGFC expression was increased in both cell lines with c‐Myc overexpression compared with the cells infected with vector control. The downstream targets of c‐Myc, such as E2F1 and TERT,^20^, ^21^ were also shown to be positively correlated with c‐Myc levels, as presented in Figure S2. The results suggest that VEGFC expression is positively correlated with c‐Myc activation. In addition, the VEGFC secretion was increased in both cell lines with c‐Myc overexpression, as shown in Figure 1C. The results demonstrate that c‐Myc upregulates VEGFC expression in pNET cells.

### 3.2 c‐Myc induces VEGFC expression via transcriptional upregulation

**FIGURE 2:**
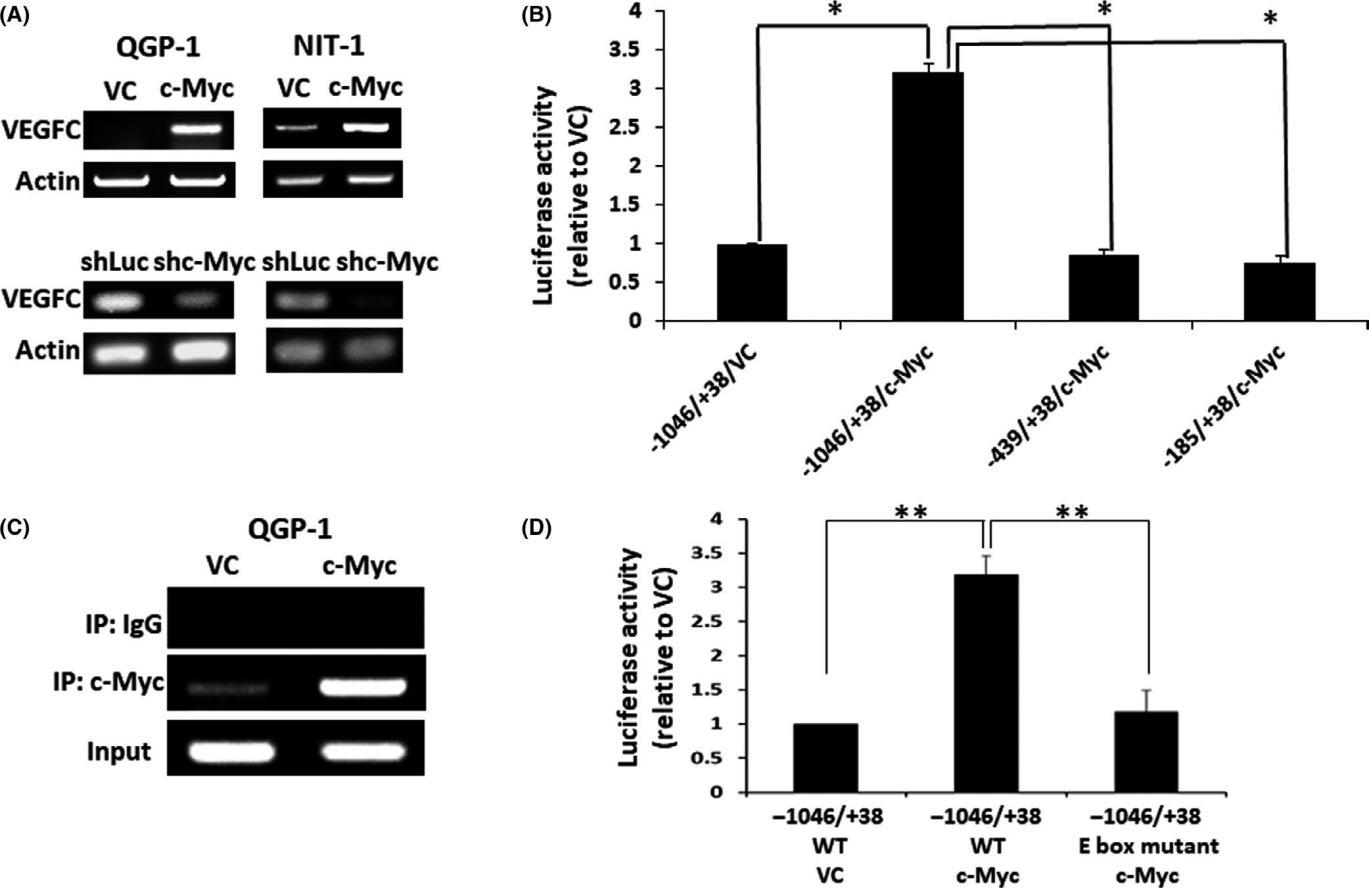
c‐Myc transcriptionally upregulates vascular endothelial growth factor C (VEGFC) expression. A, RNA expression of QGP‐1 and NIT‐1 cells with and without c‐Myc overexpression or knockdown by RT‐PCR. VC, vector control. B, The luciferase activity of the VEGFC promoter in 293T cells transfected with three VEGFC promoter plasmids (−1046/+38, −439/+38, −185/+38) with stimulation of condition medium from QGP‐1 cells with and without c‐Myc overexpression. *P < .01. C, Chromatin immunoprecipitation (ChIP) assay for VEGFC and c‐Myc in QGP‐1 cells with and without c‐Myc overexpression. D, Luciferase activity of the VEGFC promoter in 293T cells transfected with wild‐type or E‐box–mutant VEGFC promoter (−1046/+38) and stimulation of condition medium from QGP‐1 cells with or without c‐Myc overexpression. **P < .01

We further evaluated whether c‐Myc regulates VEGFC expression at transcriptional level. Figure 2A shows that c‐Myc overexpression increased the mRNA expression of VEGFC in both QGP‐1 and NIT‐1 cells. By contrast, knockdown of c‐Myc reduced the mRNA expression of VEGFC in QGP‐1 and NIT‐1 cells. Because c‐Myc is a transcriptional factor, we further investigated whether c‐Myc directly binds to the VEGFC promoter. There is an E‐box, which is a binding site for c‐Myc, located at −904 to −900 of the VEGFC promoter according to PROMO, a program to predict transcription factor–binding sites in DNA sequences.^22^ Because the VEGFC promoter plasmid was difficult to transfect into QGP‐1 cells, we alternatively performed promoter assay in 293T cells. We constructed three plasmids containing specified lengths of the promoter of VEGFC (−1046/+38, −439/+38, and −185/+38) with luciferase reporter and transfected the plasmids into 293T cells with or without c‐Myc overexpression. The VEGFC promoter activity was enhanced in 293T cells with c‐Myc overexpression at the transfected plasmid containing the full length (−1046/+38), as shown in Figure 2B. The VEGFC promoter activity was not enhanced when the transfected plasmid was deleted (−439/+38 and −185/+38). The result suggests that the −1046 to −439 promoter region of VEGFC contains the c‐Myc–regulating element. Then, we found that an enriched PCR signal specific for the E‐box was noted in QGP‐1 cells with c‐Myc overexpression by ChIP assay, as shown in Figure 2C. The VEGFC promoter activity was abolished when the E‐Box sequence was mutated, as shown in Figure 2D. Moreover, we checked the expression of MAX, a coactivator of c‐Myc,^23^ in QGP‐1 cells with overexpression or knockdown of c‐Myc and their controls by immunoprecipitation with c‐Myc. The result showed that MAX expression was present and correlated with the expression of c‐Myc, but it was not present in the lysates precipitated with IgG control (Figure S3). The expression of acetyl‐histone 3 (K9/K14), which is a histone marker for gene transcription,^24^ was also shown in a c‐Myc–dependent manner (Figure S2). As MAX is a prerequisite for specific binding to DNA at the E‐box,^25^ the result confirmed the dimerization of c‐Myc with MAX and the binding of c‐Myc to the E‐box of the VEGFC promoter. The results demonstrate that c‐Myc transcriptionally upregulates VEGFC expression through directly binding to the E‐box of the VEGFC promoter.

### 3.3 c‐Myc enhances tube formation of LECs via induction of VEGFC/VEGFR3 interaction

**FIGURE 3:**
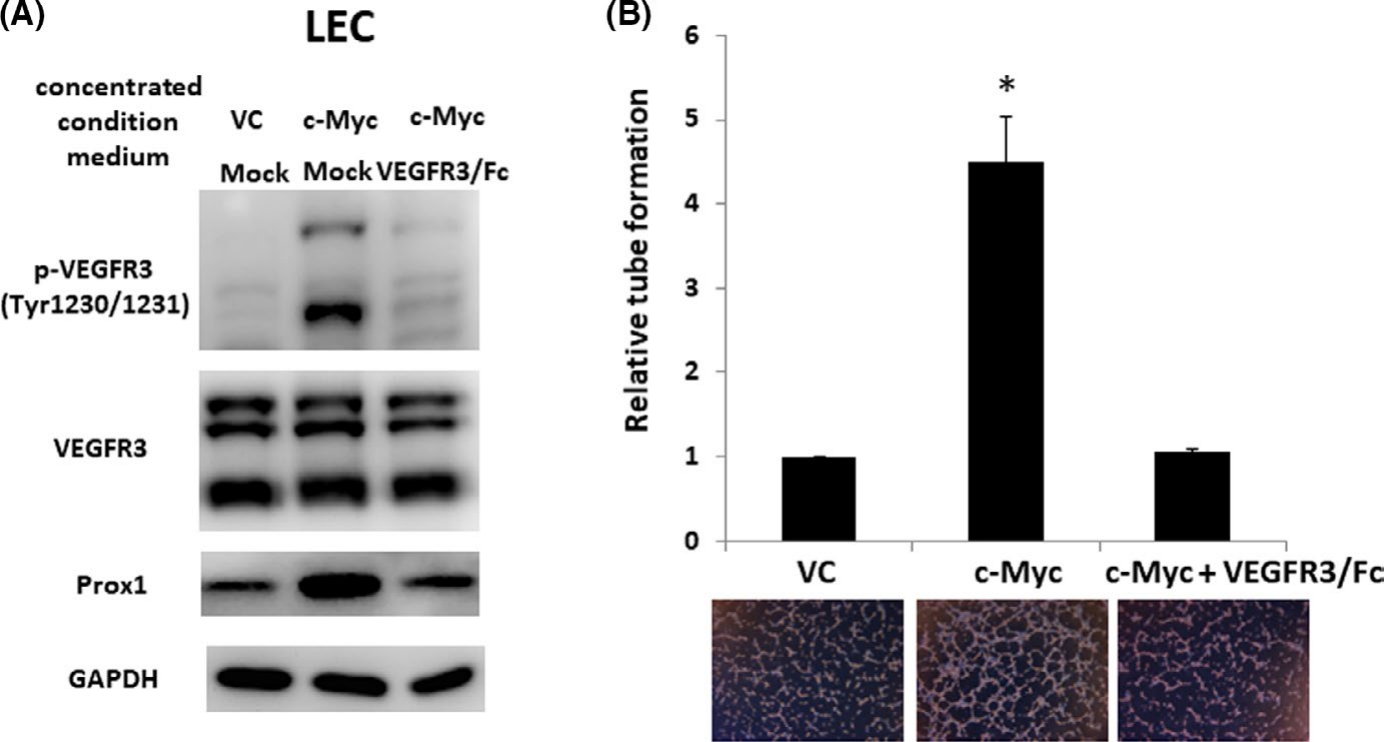
c‐Myc enhanced vascular endothelial growth factor receptor 3 (VEGFR3) phosphorylation and tube formation of lymphoepithelial cell (LECs). A, Expression of phosphorylated VEGFR3 and Prox1 in LECs with the addition of condition medium derived from QGP‐1 cells with and without c‐Myc overexpression and the addition of VEGFR3/Fc in the LEC culture in condition medium from QGP‐1 cells with c‐Myc overexpression. B, Tube formation of LECs treated with condition medium in the three conditions of (A). Relative tube formation of LECs treated with condition medium derived from QGP‐1 cells without c‐Myc overexpression (vector control [VC]) was defined as 1. *P < .05

The main function of VEGFC is to trigger lymphangiogenesis via its receptor VEGFR3. We added condition medium derived from QGP‐1 cells with or without c‐Myc overexpression into LECs. Figure 3A shows that increased VEGFR3 phosphorylation and Prox1, the downstream target of VEGFR3,^26^ were noted in LECs to which condition medium derived from QGP‐1 cells with c‐Myc overexpression was added, compared with vector control. Moreover, the VEGFR3 phosphorylation and Prox1 were reduced when coadministered with VEGFR3/Fc chimera protein, a VEGFC‐neutralizing recombinant protein. Furthermore, enhanced tube formation was noted in LECs administered with the condition medium derived from QGP‐1 cells with c‐Myc overexpression, as shown in Figure 3B. The enhanced tube formation in LECs in such a condition was suppressed by the addition of VEGFR3/Fc chimera protein. The results demonstrate that c‐Myc promotes tube formation of LECs via inducing VEGFC secretion and then activating VEGFR3 signaling.

### 3.4 c‐Myc promotes lymph node metastasis in a xenograft mouse model

**FIGURE 4:**
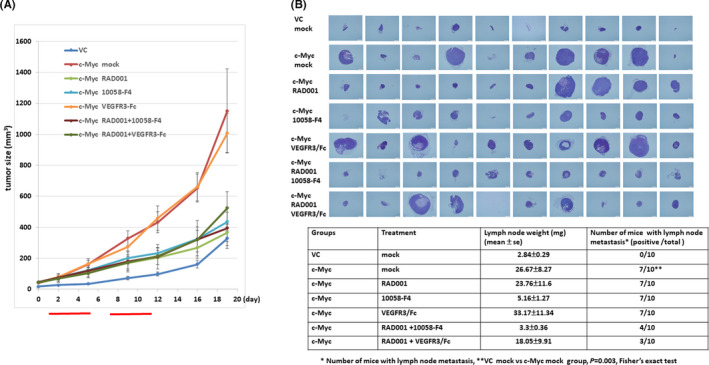
The effect of mTOR inhibitor, c‐Myc inhibitor, and vascular endothelial growth factor C (VEGFC)‐neutralizing recombinant protein on tumor growth and lymph node metastasis in a pancreatic neuroendocrine tumor (pNET) mouse model. A, Tumor growth curves of c‐Myc–overexpressing QGP‐1 xenograft mice treated with RAD001, 10058‐F4, VEGFR3/Fc, or a combination of RAD001 and 10058‐F4 or VEGFR3/Fc (vector control [VC] vs c‐Myc overexpression group,*P* = .003; c‐Myc overexpression vs c‐Myc overexpression + RAD001,*P* = .005; c‐Myc overexpression vs c‐Myc overexpression + 10058‐F4,*P* = .009; c‐Myc overexpression vs c‐Myc overexpression + RAD001 + 10058‐F4,*P* = .01; c‐Myc overexpression vs c‐Myc overexpression + RAD001 + VEGFR3/Fc,*P* = .02, Wilcoxon’s rank‐sum test). B, H&amp;E staining of the lymph node of mice in each group (upper) and the weight (mean ± standard error) and number of mice with lymph node metastasis in each group

We used a QGP‐1 xenograft mouse model to investigate the effect of c‐Myc on lymphatic metastasis in vivo and to identify the potential target to block the lymphatic metastasis of pNET. QGP‐1 cells with and without c‐Myc overexpression were subcutaneously injected into NOD‐SCID mice, and the tumor growth was observed. The mice inoculated with QGP‐1 cells with c‐Myc overexpression were treated with vehicle, RAD001 (mTOR inhibitor), 10058‐F4 (c‐Myc inhibitor), and VEGFR3/Fc (VEGFC‐neutralizing recombinant protein) alone or a combination of RAD001 and 10058‐F4 or VEGFR3/Fc for 2 weeks. There were 10 mice in each group. The gross picture of tumor in each mouse and the presence of lymph node metastasis in each mouse at the end of the in vivo experiment are shown in Figure S4 and Table S1. Figure 4A shows that c‐Myc overexpression promoted tumor growth of pNET cells (vector control vs c‐Myc overexpression group, *P* = 0.003, Wilcoxon’s rank‐sum test). The enhanced tumor growth can be demonstrated by significant increase in Ki‐67 staining of the tumors in the c‐Myc overexpression group (median 22.5%, range 5%‐45%) compared with that in the vector control group (median 3.5%, range 2%‐10%; *P* = .002, Wilcoxon’s rank‐sum test; Table S2). The tumor growth in mice with c‐Myc overexpression was suppressed by treatment with RAD001 (*P* = .005) or 10058‐F4 (*P* = .009) alone or a combination of RAD001 and 10058‐F4 (*P* = .01) or VEGFR3/Fc (*P* = .02). We excised the proximal lymph node (inguinal area) of all mice and the weight of lymph node was measured for each mouse. H&E staining of the lymph node was performed to determine the positivity of lymph node metastasis in each mouse, as shown in Figure 4B. The size of lymph nodes in the mice with c‐Myc overexpression and treated with 10058‐F4 (5.16 ± 1.27 mg) or a combination of RAD001 and 10058‐F4 (3.3 ± 0.36 mg) was decreased compared with the mice with c‐Myc overexpression without treatment (mock control; 26.67 ± 8.27 mg). Microscopically, there was no lymph node structure noted in one mouse in the vector, c‐Myc overexpression + 10058‐F4, and c‐Myc overexpression + RAD001 + VEGFR3/Fc group, respectively. There was no lymph node metastasis in the control group, but seven mice in the c‐Myc overexpression group had lymph node metastasis (*P* = .003, Fisher's exact test). However, RAD001, 10058‐F4, or VEGFR3/Fc alone did not reduce the number of mice with lymph node metastasis. Combination of RAD001 with 10058‐F4 (*P* = .37, Fisher's exact test) or VEGFR3/Fc (*P* = .18, Fisher's exact test) tended to reduce lymph node metastasis in mice with c‐Myc overexpression , although it is not statistically significant. We evaluated the expression of VEGFC in the tumors in each group by immunohistochemical staining. The percentages and intensities of VEGFC expression in the tumors of the mice in the vector control and c‐Myc overexpression group are shown in Table S2. There were three mice with high expression of VEGFC in the control group, whereas nine mice had high expression of VEGFC in the group with c‐Myc overexpression (*P* = .02, Fisher's exact test). The VEGFC expression was increased by c‐Myc overexpression in QGP‐1 xenograft mice. We stained LYVE1 to evaluate the LVD in the tumors in each group. The median intratumor LVD in each mouse is shown in Table S3. The median intratumor LVD in the control group and c‐Myc overexpression group were 2.0 and 2.5, respectively (*P* = .56, Wilcoxon’s rank‐sum test). When the intratumor LVD in the c‐Myc–overexpressed mice treated with the indicated drugs was compared with that in the c‐Myc–overexpressed group without treatment (mock), there was a trend of reduced intratumor LVD in the group treated with a combination of RAD001 and 10058‐F4 (c‐Myc overexpression + mock vs c‐Myc overexpression + RAD001 + 10058‐F4, median LVD 2.5 vs 0, *P* = .11) or VEGFR3/Fc (c‐Myc overexpression + mock vs c‐Myc overexpression + RAD001 + VEGFR3/Fc, median LVD 2.5 vs 0, *P* = .1), although not statistically significant. A representative figure of VEGFC, Ki‐67, and LYVE1 in the vector control and c‐Myc overexpression is shown in Figure S5. The results suggest that c‐Myc promotes lymphangiogenesis and lymph node metastasis in pNET.

The status of c‐Myc expression^17^ and the presence of lymph node metastasis of 21 pNET patients in our previous study are shown in Table S4. Six of the 17 patients had lymph node metastasis, and all of them had high expression of c‐Myc. All four patients with low expression of c‐Myc did not have lymph node metastasis. The association of c‐Myc expression with lymph node metastasis was not significant in our pNET samples (*P* = .28), possibly due to the limited case number.

### 3.5 mTOR inhibitor inhibits lymphangiogenic properties of lymphatic endothelial cells

**FIGURE 5:**
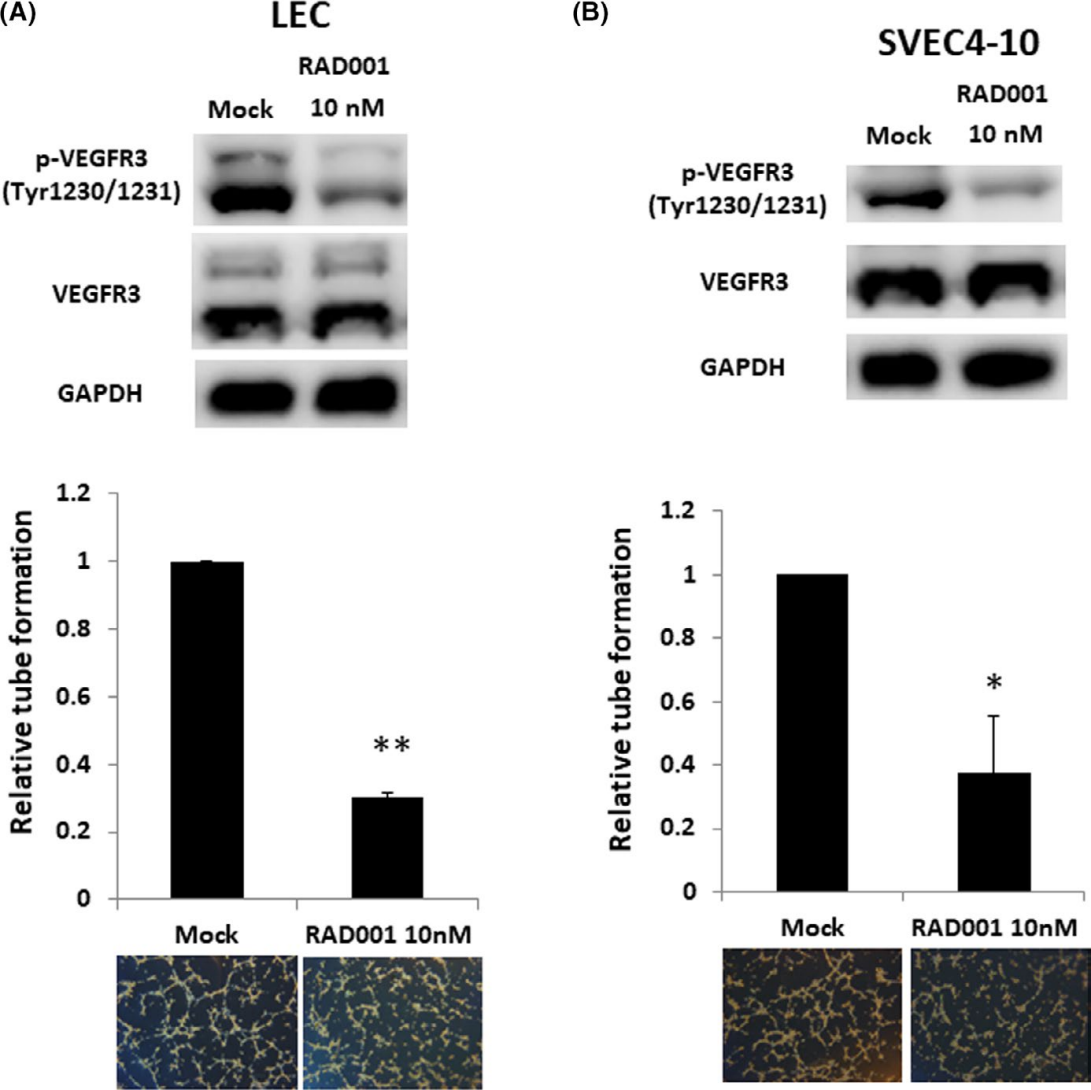
mTOR inhibitor inhibits the phosphorylation of vascular endothelial growth factor receptor 3 (VEGFR3) and tube formation of human and murine lymphoepithelial cells (LECs). A, Phosphorylated VEGFR3 expression (upper) and tube formation (lower) in human LECs treated with and without RAD001. **P < .001. B, Phosphorylated VEGFR3 expression (upper) and tube formation (lower) in murine LECs (SVEC4‐10) treated with and without RAD001. *P = .03

In QGP‐1 xenograft mice with c‐Myc overexpression, lymph node metastasis was reduced in the group treated with a combination of RAD001 and 10058‐F4 or VEGFR3/Fc but not in the group treated with RAD001, 10058‐F4, or VEGFR3/Fc alone. The result suggests that mTOR plays an important role for lymph node metastasis in pNET. We then evaluated the effect of the mTOR inhibitor on lymphangiogenesis. Figure 5A shows that RAD001 reduced the phosphorylation of VEGFR3 and the tube formation of LECs. Similar result was also presented in murine LECs (SVEC4‐10), as shown in Figure 5B, which was consistent with the result in an animal model. RAD001 also reduced the expression of c‐Myc and VEGFC in QGP‐1 cells, as shown in Figure S6. The results suggest that the mTOR inhibitor suppresses lymphangiogenesis in pNET via inhibiting the VEGFC expression of pNET cells and direct effect on the tube formation of LECs.

## 4 DISCUSSION

In this study, we demonstrate that c‐Myc overexpression transcriptionally upregulates VEGFC expression in pNET cells, which promotes lymphangiogenesis of pNET cells in vitro and in vivo. VEGFC is a member of the PDGF family.^27^ It is indispensable for embryonic and adult lymphangiogenesis,^28^ and it correlates significantly with lymphangiogenesis and the proliferation and migration of vascular endothelial cells.^27^ VEGFC is produced by macrophage and granulocytes and promotes lymphangiogenesis at sites of tissue inflammation.^29^, ^30^ However, VEGFC can also be secreted by cancer cells themselves and directly promote cancer cell migration and invasion, tumor‐associated lymphangiogenesis, and lymphatic metastasis.^8^, ^31^, ^32^ VEGFC and VEGFD bind to VEGFR3 and activate the downstream signals, which may induce lymphangiogenesis and promote lymph node and organ metastasis in various cancers.^28^, ^33^, ^34^ VEGFC can be transcriptionally regulated by cytokines (interleukin‐1α, interleukin‐1β, or tumor necrosis factor‐α) or growth factors (PDGF, EGF, TGF‐β).^35^, ^36^ In addition, micro RNAs (miR27b, miR‐101, and miR‐128) have been shown to downregulate VEGFC expression and lead to the suppression of tumor growth, metastasis, invasion, migration, and angiogenesis in gastric cancer, bladder cancer, and hepatocellular carcinoma.^37^, ^38^, ^39^ CCL5 has also been shown to promote VEGFC production and induce lymphangiogenesis via suppressing miR‐507, which binds to 3′UTR of the *VEGFC* gene, in human chondrosarcoma cells.^40^ In an Eμ‐c‐Myc transgenic mouse model, a highly metastatic murine model of Burkitt's lymphoma, c‐Myc was shown to stimulate angiogenesis and lymphangiogenesis accompanied with increased expression of VEGF by immunohistochemical staining.^41^ However, the causal effect of c‐Myc with VEGF was not understood. We demonstrated that c‐Myc is a transcriptional factor that binds to the promoter of VEGFC and promotes the transcription and secretion of VEGFC in QGP‐1 cells. In our previous study, we demonstrated that PTEN and LKB1 transcriptionally regulate c‐Myc expression, whereas c‐Myc can negatively backregulate PTEN expression and positively regulate the expression of the downstream signals of mTOR to promote cell proliferation and attenuate the sensitivity of pNET cell lines to the mTOR inhibitor in vitro and in vivo. In that study, 11 (52%) of 21 pNET patients had low expression of PTEN and/or LKB1 in their tumor samples but 17 (81%) had high expression of c‐Myc. High expression of c‐Myc can also be present in pNET without loss of PTEN and/or LKB1.^17^ Negative PTEN expression has been shown to be significantly associated with lymph node metastases in breast cancer and non–small cell lung cancer patients.^42^, ^43^ However, c‐Myc can be regulated by many molecules other than PTEN and LKB1.^44^ The result warrants further investigation regarding c‐Myc regulation by a PTEN/LKB1‐independent mechanism. Regardless of the regulatory mechanism of c‐Myc in pNET, our current study suggests that c‐Myc and VEGFC are potential targets for the inhibition of lymphangiogenesis and lymph node metastasis in pNET.

In our animal model, we have shown that c‐Myc promoted tumor growth and lymph node metastasis in QGP‐1 xenograft mice. The tumor growth in QGP‐1 xenograft mice with c‐Myc overexpression could be suppressed by RAD001 or the c‐Myc inhibitor alone but not by the VEGFC‐neutralizing recombinant protein. The result suggests that mTOR and c‐Myc, but not VEGFC, were involved in tumor growth. c‐Myc promotes lymph node metastasis, and the size of lymph node was reduced in the mice treated with the c‐Myc inhibitor in our animal study (Figure 4B). However, the number of mice with lymph node metastasis was not significantly reduced by treatment with the c‐Myc inhibitor or VEGFC‐neutralizing recombinant protein alone, but it was reduced by treatment with a combination of the mTOR inhibitor and one of them. The result suggests that mTOR in addition to c‐Myc is also an important signal for lymphangiogenesis in pNET. Activation of mTOR pathway was shown to be associated with increased LVD and lymph node metastasis via upregulation of VEGFC in various cancers in in vitro and in vivo models and in human samples.^45^, ^46^, ^47^ mTOR inhibitors decreased VEGFC/D expression and diminished lymphangiogenesis and lymph node metastasis in various cancer models.^45^, ^46^, ^47^, ^48^ The results demonstrate the role of mTOR activation in lymphangiogenesis by VEGFC in LECs and primary tumors. In our study, RAD001 was shown to decrease the VEGFC expression of QGP‐1 cells (Figure S6). RAD001 suppressed VEGFR3 phosphorylation and inhibited the tube formation of human and murine LECs, as shown in Figure 5. The results suggest that the mTOR inhibitor targets both tumor cells and LECs for inhibition of lymphangiogenesis in pNET.

**FIGURE 6:**
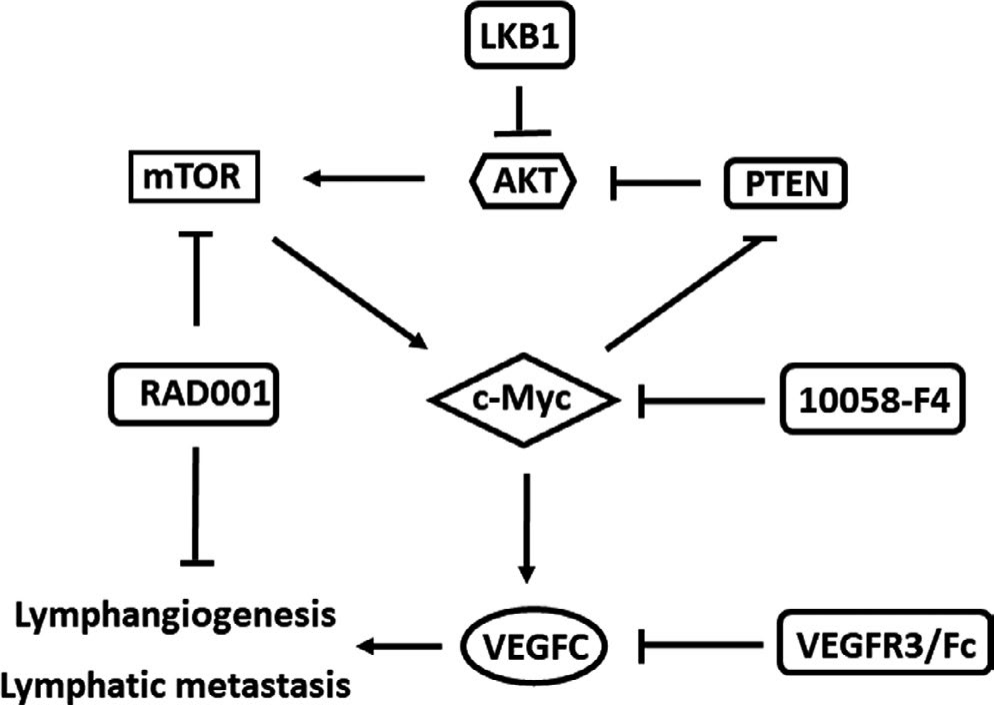
The putative model of lymphatic metastasis in pancreatic neuroendocrine tumor (pNET). c‐Myc can be regulated by PTEN and LKB1 via the AKT/mTOR axis, and it can backregulate PTEN to activate the mTOR pathway. c‐Myc can upregulate VEGFC expression and the secretion of QGP‐1 cells (pNET) and promotes lymphangiogenesis and lymph node metastasis. mTOR is constitutively activated in pNET and can be negatively regulated by PTEN and/or LKB1. mTOR promotes lymphangiogenesis in pNET via increasing vascular endothelial growth factor C (VEGFC) expression of pNET cells and inhibiting tube formation of lymphoepithelial cells (LECs) in pNET. Combined treatment with an mTOR inhibitor, RAD001, and a c‐Myc inhibitor (10058‐F4) or a VEGFC‐neutralizing chimeric protein (VEGFR/Fc) reduces the lymphangiogenesis and lymph node metastasis of pNET in a mouse model

Activation of the mTOR pathway has been shown in pNET according to gene expression array and immunohistochemistry.^49^, ^50^ The association between mTOR and c‐Myc in pNET has been shown in our previous study.^17^ In the current study, we have delineated the regulatory mechanism of lymphangiogenesis and lymph node metastasis by c‐Myc in pNET. Abrogating the effect of VEGFC on LECs by a c‐Myc inhibitor or VEGFC‐neutralizing recombinant protein may reduce the tube formation of LECs. On the other hand, inhibition of mTOR by an mTOR inhibitor may decrease the VEGFC expression of pNET cells and reduce the tube formation of LECs directly. In our animal study, the decreasing trend of lymph node metastasis was only observed in a combination of the mTOR inhibitor with the VEGFC‐neutralizing recombinant protein or c‐Myc inhibitor. Taken together, our current and previous studies suggest that PTEN and/or LKB1 negatively activate the mTOR pathway and c‐Myc expression, and both mTOR and c‐Myc are important for lymphangiogenesis and lymph node metastasis in pNET. Our studies have made a link showing the association of PTEN and LKB1 with mTOR and c‐Myc. The putative model is shown in Figure 6.

In conclusion, c‐Myc promotes lymphatic metastasis via transcriptional upregulation of VEGFC in pNET, whereas mTOR activation is also important for the lymphangiogenesis of pNET. Combined targeting of mTOR and c‐Myc/VEGFC is a potential therapy for prevention and treatment of lymphatic metastasis in pNET.

## CONFLICT OF INTEREST

The authors disclosed no financial conflict of interests.

## Supporting information

Fig S1

Fig S2

Fig S3

Fig S4

Fig S5

Fig S6

Table S1

Table S2

Table S3

Table S4
