# Supplementary figures and images for: c‐Myc promotes lymphatic metastasis of pancreatic neuroendocrine tumor through VEGFC upregulation
Source: Cancer Sci. 2020 Nov 24;112(1):243–53. doi: 10.1111/cas.14717 (PMC7780026; doi:10.1111/cas.14717)

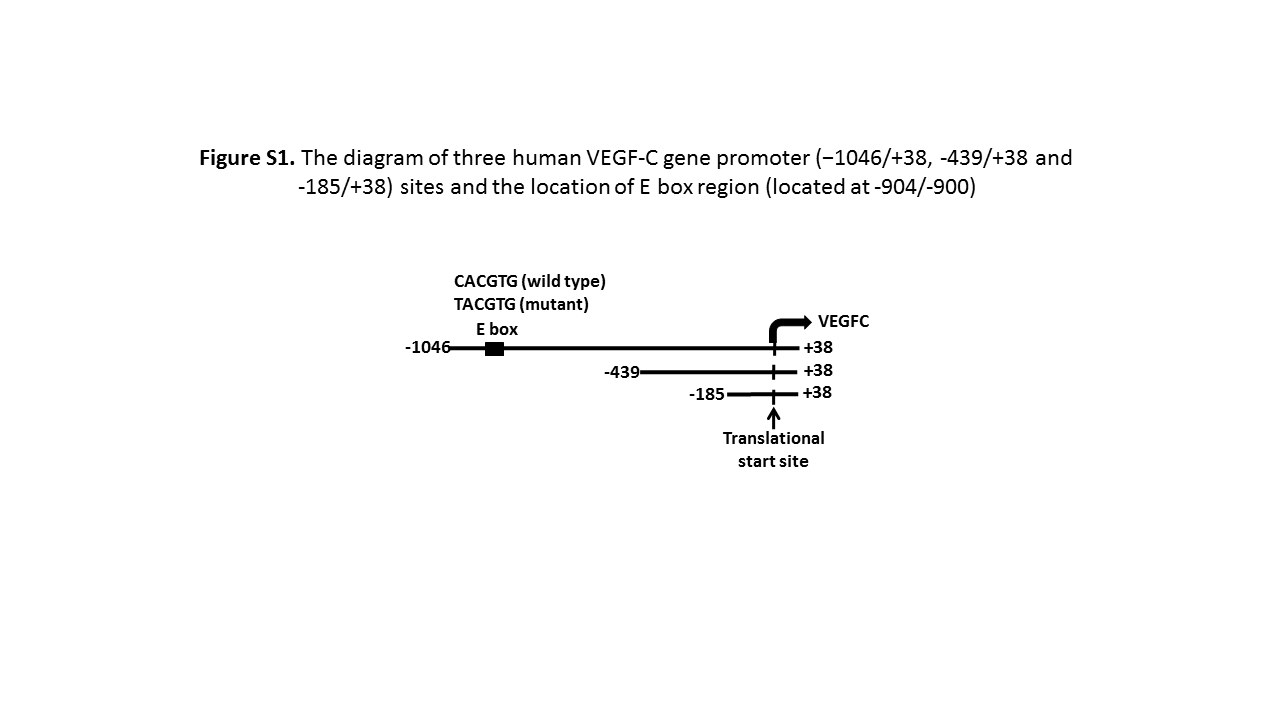

Supplement: Supplementary file 1 — Fig S1 [file CAS-112-243-s001.tif]

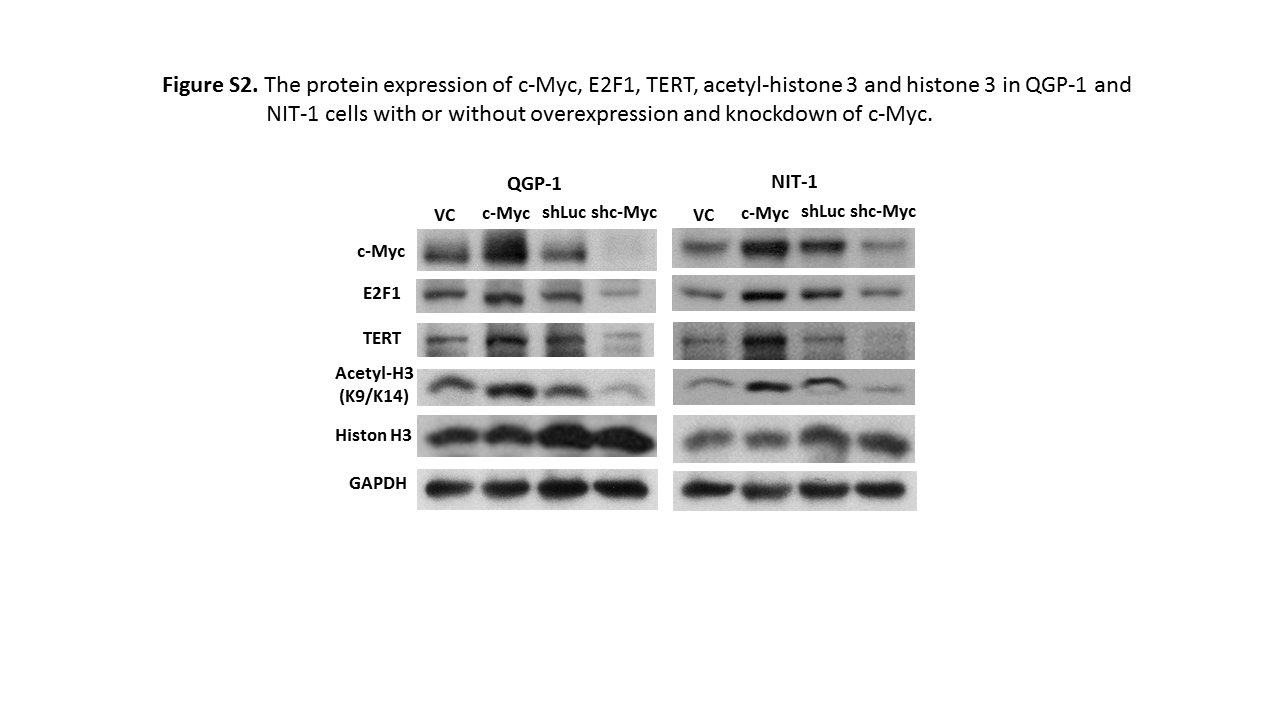

Supplement: Supplementary file 2 — Fig S2 [file CAS-112-243-s002.tif]

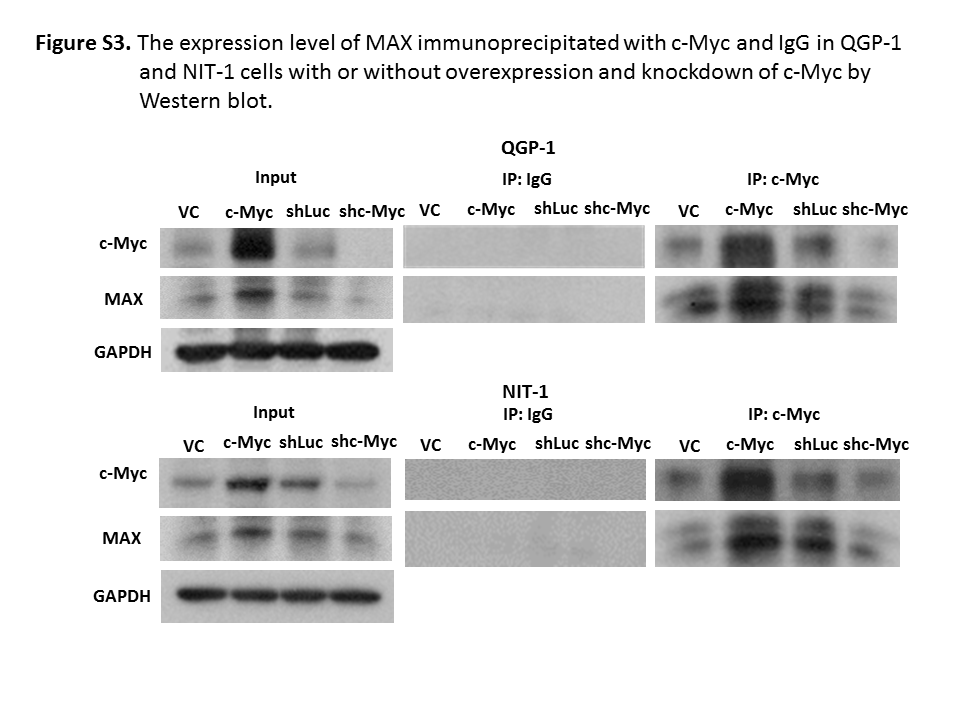

Supplement: Supplementary file 3 — Fig S3 [file CAS-112-243-s003.tif]

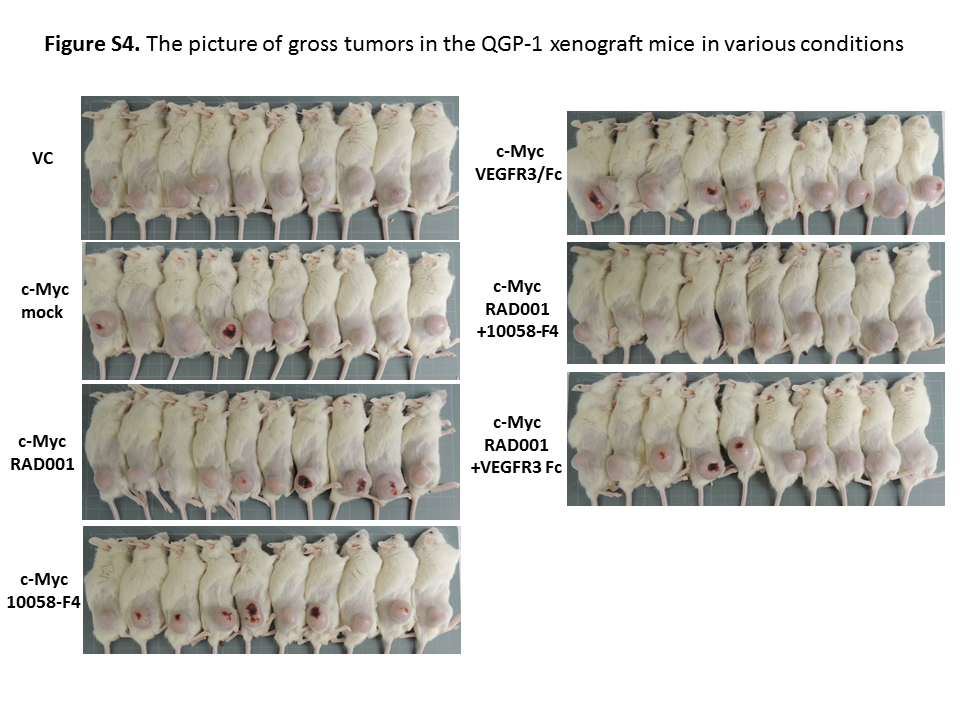

Supplement: Supplementary file 4 — Fig S4 [file CAS-112-243-s004.tif]

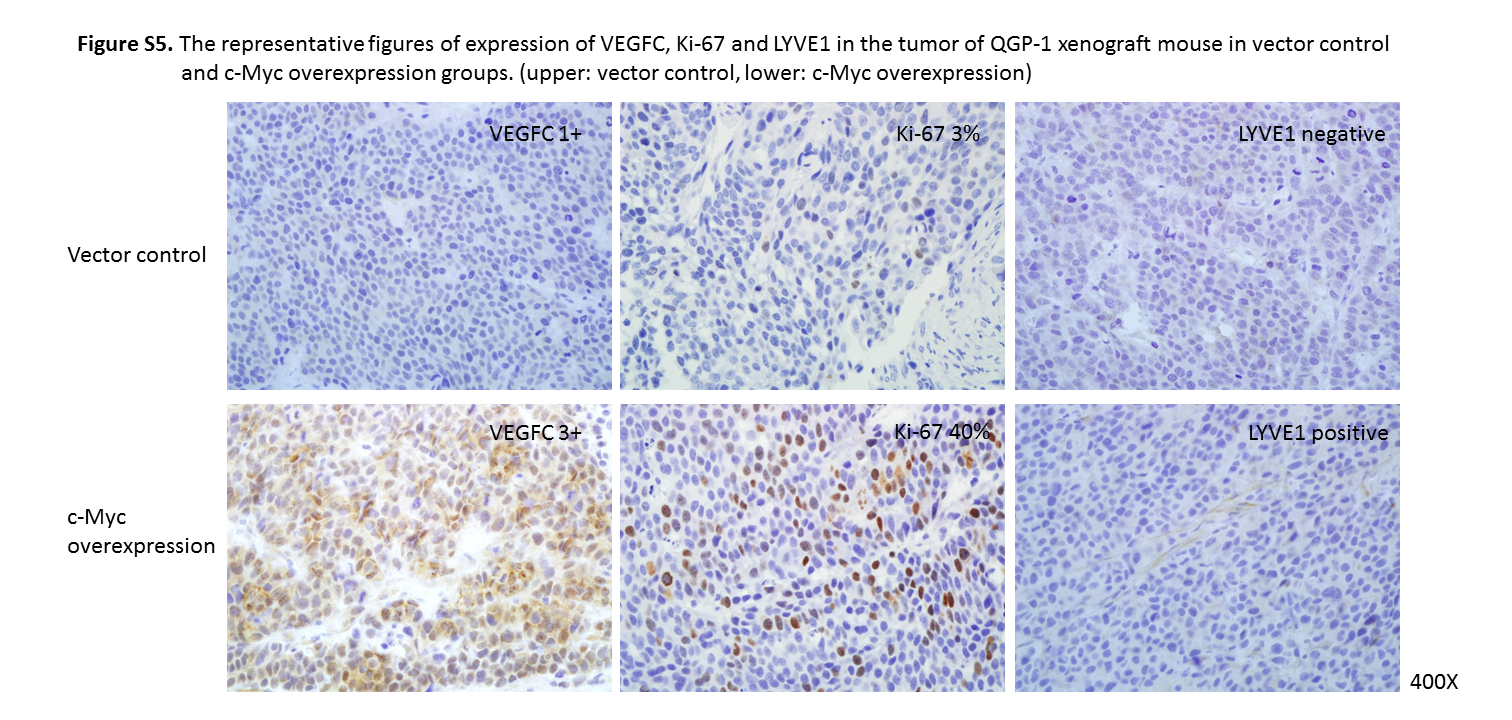

Supplement: Supplementary file 5 — Fig S5 [file CAS-112-243-s005.tif]

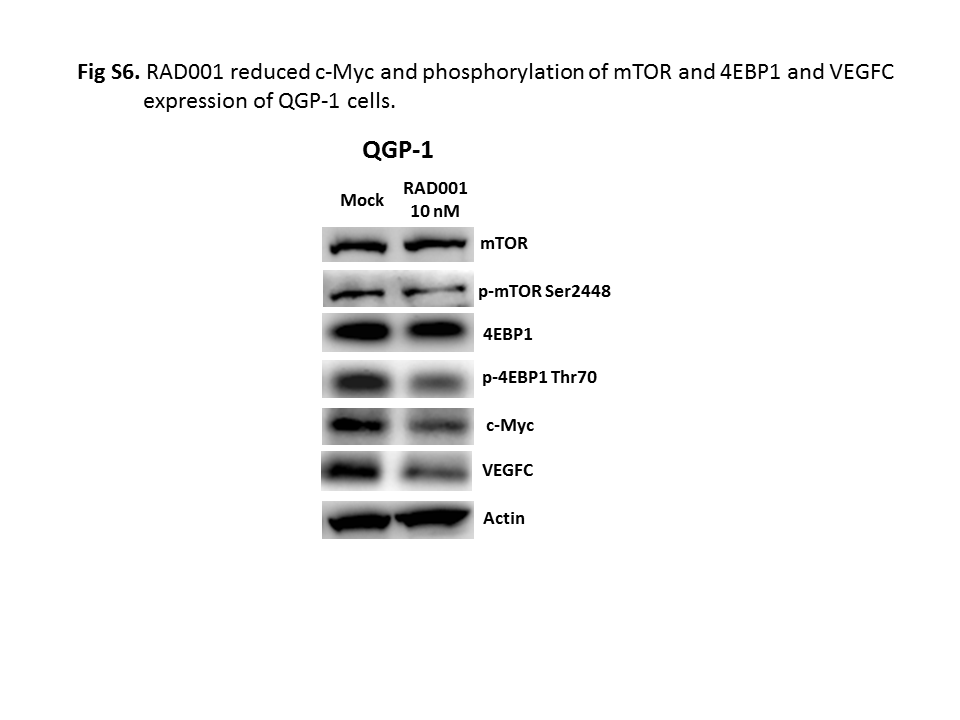

Supplement: Supplementary file 6 — Fig S6 [file CAS-112-243-s006.tif]
