# Supplementary material for: c‐Myc promotes lymphatic metastasis of pancreatic neuroendocrine tumor through VEGFC upregulation
Source: Cancer Sci. 2020 Nov 24;112(1):243–53. doi: 10.1111/cas.14717 (PMC7780026; doi:10.1111/cas.14717)
Supplement: Supplementary file 7 — Table S1 [file CAS-112-243-s007.docx]

Table S1. The status of presence of lymph node metastasis in each one of QGP-1 xenograft mice.

| group | regional lymph node metastasis | | | | | | | | | |
| --- | --- | --- | --- | --- | --- | --- | --- | --- | --- | --- |
| VC  (vector control) | - | - | - | - | - | no lymph node structure | - | - | - | - |
| c-Myc* +  mock | + | - | - | + | + | + | + | + | + | - |
| c-Myc* + RAD001 | - | - | + | - | + | + | + | + | + | + |
| c-Myc* +  10058-F4 | no lymph node structure | + | + | + | + | - | + | + | + | - |
| c-Myc* + VEGFR3/Fc | + | - | + | + | + | - | + | + | + | - |
| c-Myc*+ RAD001 + 10058-F4 | - | - | - | - | + | + | + | - | - | + |
| c-Myc* + RAD001 + VEGFR3/Fc | - | - | + | + | no lymph node structure | - | + | - | - | - |

*, c-Myc overexpression
