# Supplementary material for: c‐Myc promotes lymphatic metastasis of pancreatic neuroendocrine tumor through VEGFC upregulation
Source: Cancer Sci. 2020 Nov 24;112(1):243–53. doi: 10.1111/cas.14717 (PMC7780026; doi:10.1111/cas.14717)
Supplement: Supplementary file 8 — Table S2 [file CAS-112-243-s008.docx]

Table S2. The percentage and intensity of VEGFC expression, intratumor LVD and Ki-67 expression in the tumor of each mouse in vector control and c-Myc overexpression groups.

|  | VEGFC intensity and percentage (%) | | | | Intratumor LVD | Ki-67 (%) |
| --- | --- | --- | --- | --- | --- | --- |
|  | 3+ | 2+ | 1+ | 0* |  |  |
| VC  (vector control) | 0 | 10 | 85 | 5 | 5 | 5 |
|  | 0 | 5 | 90 | 5 | 2 | 4 |
|  | 0 | 0 | 45 | 55 | 4 | 2 |
|  | 0 | 0 | 25 | 75 | 3 | 3 |
|  | 0 | 0 | 60 | 40 | 0 | 2 |
|  | 0 | 0 | 95 | 5 | 3 | 5 |
|  | 0 | 10 | 90 | 0 | 2 | 3 |
|  | 0 | 0 | 35 | 65 | 0 | 3 |
|  | 0 | 0 | 100 | 0 | 0 | 4 |
|  | 0 | 0 | 40 | 60 | 0 | 10 |
| c-Myc overexpression | 0 | 10 | 60 | 30 | 5 | 30 |
|  | 0 | 5 | 30 | 65 | 0 | 5 |
|  | 0 | 25 | 30 | 45 | 0 | 10 |
|  | 5 | 20 | 45 | 30 | 2 | 10 |
|  | 20 | 25 | 45 | 10 | 0 | 40 |
|  | 0 | 5 | 40 | 55 | 5 | 25 |
|  | 0 | 0 | 55 | 45 | 5 | 40 |
|  | 0 | 10 | 30 | 60 | 3 | 15 |
|  | 0 | 10 | 50 | 40 | 5 | 45 |
|  | 5 | 15 | 45 | 35 | 0 | 20 |
| *P* value  (VC vs c-Myc overexpression) | 0.02** | | | | 0.56! | 0.002! |

*,0: no expression

**, Fisher’s exact test

!, Wilcoxon Rank-Sum test
