# Supplementary material for: c‐Myc promotes lymphatic metastasis of pancreatic neuroendocrine tumor through VEGFC upregulation
Source: Cancer Sci. 2020 Nov 24;112(1):243–53. doi: 10.1111/cas.14717 (PMC7780026; doi:10.1111/cas.14717)
Supplement: Supplementary file 9 — Table S3 [file CAS-112-243-s009.docx]

Table S3. The intratumor lymphatic microvessel density (LVD) (number/10 high power field (200x) in the tumor areas) in each one of QGP-1 xenograft mice.

| group | Intratumor LVD | | | | | | | | | | mean | median | Fisher’s exact test  (versus c-Myc + mock) |
| --- | --- | --- | --- | --- | --- | --- | --- | --- | --- | --- | --- | --- | --- |
| VC  (vector control) | 5 | 2 | 4 | 3 | 0 | 3 | 2 | 0 | 0 | 0 | 1.9 | 2 | 0.56 |
| c-Myc* +  mock | 5 | 0 | 0 | 2 | 0 | 5 | 5 | 3 | 5 | 0 | 2.5 | 2.5 | - |
| c-Myc* +  RAD001 | 0 | 0 | 0 | 2 | 0 | 3 | 2 | 2 | 1 | 5 | 1.5 | 1.5 | 0.73 |
| c-Myc* +  10058-F4 | 0 | 4 | 3 | 2 | 3 | 3 | 5 | 0 | 0 | 0 | 2 | 2.5 | 0.61 |
| c-Myc* + VEGFR3/Fc | 0 | 0 | 4 | 2 | 0 | 3 | 2 | 5 | 0 | 0 | 1.6 | 1.0 | 0.39 |
| c-Myc*+ RAD001 + 10058-F4 | 0 | 0 | 0 | 0 | 1 | 1 | 2 | 2 | 0 | 0 | 0.6 | 0 | 0.11 |
| c-Myc* + RAD001 + VEGFR3/Fc | 0 | 0 | 0 | 2 | 4 | 0 | 2 | 0 | 0 | 0 | 0.8 | 0 | 0.1 |

*, c-Myc overexpression
