# Supplementary material for: c‐Myc promotes lymphatic metastasis of pancreatic neuroendocrine tumor through VEGFC upregulation
Source: Cancer Sci. 2020 Nov 24;112(1):243–53. doi: 10.1111/cas.14717 (PMC7780026; doi:10.1111/cas.14717)
Supplement: Supplementary file 10 — Table S4 [file CAS-112-243-s010.docx]

Table S4. The expression of c-Myc and status of lymph node metastasis in pNET patients

| **Case No.** | **c-Myc** | **LN metastasis** |
| --- | --- | --- |
| 1 | 2+ | - |
| 2 | 2+ | - |
| 3 | 2+ | - |
| 4 | 2+ | - |
| 5 | 2+ | + |
| 6 | 2+ | + |
| 7 | 1+ | - |
| 8 | 2+ | + |
| 9 | 2+ | + |
| 10 | 3+ | - |
| 11 | 3+ | + |
| 12 | 2+ | + |
| 13 | 1+ | - |
| 14 | 2+ | - |
| 15 | 3+ | - |
| 16 | 1+ | - |
| 17 | 3+ | - |
| 18 | 3+ | - |
| 19 | 2+ | - |
| 20 | 2+ | - |
| 21 | 1+ | - |

|  | lymph node metastasis - | lymph node metastasis + |
| --- | --- | --- |
| low expression of c-Myc | 4 | 0 |
| high expression of c-Myc | 11 | 6 |

The association of c-Myc expression with lymph node metastasis *P*=0.28, Fisher’s exact test.
